# Theorizing the Role of Sex Educators in the Resistance and Reification of Epistemic Injustices Related to the Sexual Expression of People with Intellectual Disability

**DOI:** 10.1007/s10508-024-03039-5

**Published:** 2024-11-14

**Authors:** Sarah L. Curtiss, Melissa Stoffers

**Affiliations:** 1 School of Education, University of Delaware, 015A Willard Hall Education Building, 16 W Main Street, Newark, DE 19716 USA; 2 Human Development and Family Sciences, University of Delaware, Newark, DE USA

**Keywords:** Intellectual disability, Sex education, Epistemic injustice, Grounded theory

## Abstract

People with intellectual disability face a variety of epistemic injustices—systems of knowledge and educational inequality—related to their access to sex education and perceptions of their sexuality. Sex educators are in a position to address these injustices; however, they may not be fully aware of their own epistemic ethics. Furthermore, there is little theoretical understanding of how sex educators provide instruction within an environment that exhibits hostility toward the sexual expression of those with intellectual disability. This grounded theory study of interviews with 58 people who taught sex education to people with intellectual disability sought to understand the role of educators in perpetuating and resisting epistemic injustice by utilizing sex education to either facilitate or restrict the sexual expression of people with disabilities. We identified four micro-contexts: centralizing capacity to consent, emphasizing risk, desexualizing (through) sex education, and acknowledging gender identity. In each micro-context, we explore mechanisms of how educators were either allied in support of autonomy (person-centered values, expertise, access to curricular resources, self-advocates as teachers and partners, subversiveness, and de-problematizing normative sexual behavior) or reified sexual stigmas (fear of sexual abuse and sexual perpetration, conscripting to asexuality, the paternalistic service system, and complex ethical dilemmas) in the context of hostility to sex education and sexual expression. These findings have implications for promoting prosocial sexual expression and access to sex education for people with intellectual disability.

**Supplementary Information:**

The online version contains supplementary material available at 10.1007/s10508-024-03039-5.

## Introduction

Comprehensive sex education programs include medically accurate instruction addressing contraception, abstinence, human development, relationships, decision-making and disease prevention (Future of Sex Education Initiative, 2012), and address cognitive, affective, and behavioral learning domains (Sexuality Information Education Council of the United States; SIECUS, 2004). As such, comprehensive sex education programs can support the development of sex positivity, healthy relationships, and ability to make informed choices (American Academy of Pediatrics, 2024) and have been associated with a number of outcomes, such as delayed intercourse, increased contraceptive use, and fewer sexual partners (SIECUS, 2004), as well as reductions in homophobia, enhanced knowledge and skill in developing healthy relationships, and reductions in child abuse and intimate partner violence (Goldfarb & Lieberman, 2021).

Unfortunately, access varies widely, with individuals from marginalized populations less likely to receive sex education (Haberland & Rogow, 2014). Individuals with intellectual disability are one such population that is frequently excluded from sex education instruction (Barnard-Brak et al., 2014; Campbell et al., 2020; Pérez-Curiel et al., 2023). Intellectual disability is a widely recognized form of disability that is permanent, present during childhood, and medically diagnosed through cognitive and adaptive functioning assessments. People with intellectual disability often need specialized instruction to learn academic and life skills and some people will always need significant support in these areas (Boat et al., 2015). Intellectual disability may be caused by known or unknown genetic and environmental factors and may be diagnosed alongside other disabilities, such as autism, cerebral palsy, and seizure disorders (Bartoshesky & Wright, 2021). Scholars have found that individuals with intellectual disability have less knowledge about sex and sexuality than their non-disabled peers (Sinclair et al., 2015).

Additionally, people with disabilities are provided few opportunities for sexual exploration and many sexual health programs do not address the needs of those with disabilities (The United Nations Population Fund, n.d.). Despite a desire to access information and engage in conversations about sexuality and relationships, individuals with intellectual disability have reported receiving limited sex education information: in the USA, students who have individualized education plans under intellectual disability and autism designations are the least likely to receive sex education of any students (Holmes et al., 2022). This lack of access to comprehensive sex education can increase the risk of experiencing sexual assault (Campbell et al., 2020): individuals with intellectual disability are significantly more likely to experience sexual assault than those without disabilities (Amborski et al., 2022).When sex education information is available, it is not comprehensive and is largely gendered and heteronormative (Frawley & Wilson, 2016). Pleasure-based sex education curriculum is uncommon in general, but is especially rare in curricula designed for individuals with disabilities (Okotie & Jolly, 2024). Although historically desexualized by society, individuals with intellectual disability are a diverse group with varied sexual identities (Kim, 2011).

Individuals with intellectual disability may experience lower levels of knowledge pertaining to sex and sexuality because they face many barriers to accessing sex education. Interventions intended to increase the sexual knowledge of individuals with intellectual disability have been successful (Chou et al., 2019). In a meta-analysis of experimental design studies providing sex education to individuals with intellectual disability, Gonzálvez et al. (2018) found sex education programming had a moderate positive effect and specifically reduced inappropriate behaviors and sexual abuse, as well as improved decision-making and social skills. This indicates that societal barriers and lack of educational access are the likely culprits for knowledge differences. For example, barriers described in interviews with healthcare providers, parents, educators, and youth with intellectual disability include a lack of institutional support or federal mandate to ensure that sex education is both provided and accessible; a lack of efficacy among healthcare providers and educators who are unsure how to provide sex education instruction; and parents infantilizing their child and assuming their child is uninterested in sex (Schmidt et al., 2021). Social stigmas that assume individuals with intellectual disability are asexual and childlike have also led to restrictions on personal privacy and access to sexual health information (Ditchman et al., 2016). Alternatively, another societal stigma that assumes that people with disabilities are hypersexual or that sexual problems are a result of disability (Brodwin & Frederick, 2010) can also lead to restrictions on information about sex and sexuality due to concerns that this information would trigger uncontrollable sexual desires (Rugoho & Maphosa, 2017).

### Epistemic Injustice, Sexual Knowledge, and Disability

Epistemic injustice refers to systems of knowledge and educational inequality in the form of hermeneutics and testimony (Fricker, 2007) and provides an important framework through which to explore the unique intersection of power and understanding that applies to sex education and people with intellectual disability. Hermeneutical injustice results when a marginalized individual’s or group’s social experiences are not reflected within or understood by the broader majority population (Fricker, 2007; Kidd & Carel, 2018). The use of hermeneutical resources, such as communal language, metaphors, and images, is critical in allowing individuals to share and understand one another’s social experiences (Kidd & Carel, 2018). For marginalized groups, a lack of these hermeneutical resources may prevent access to knowledge and communication of knowledge to those in more socially privileged groups (McKinnon, 2016).

Hermeneutical injustice may take many forms, including being unable to make sense of one’s own experience or sharing one’s understanding of an experience with others (Kidd & Carel, 2018). This may lead to exclusionary practices, such as preventing individuals from marginalized populations to serve in authoritative positions, restricting expression to those deemed legitimate by the socially privileged population, or assigning a social expectation to an individual that may not align with the individual’s personal interests or needs (Fricker, 2007; Kidd & Carel, 2018). As a marginalized population, individuals with intellectual disability may not have the hermeneutical resources to understand and communicate their experiences regarding sex and sexuality. In part, this may be due to the societal restrictions and barriers placed upon access to sex education.

The other dimension of epistemic injustice is testimonial injustice: when those in a privileged societal group give less credibility to those in the marginalized group, thus those in the marginalized group are relegated as less able to know. The status of people with intellectual disability as less credible knowers is codified in a variety of ways. For example, although people with intellectual disability have reported a need for space for prosocial sexual expression (Medina-Rico et al., 2018), individuals with intellectual disability face societal stigmas that assign social expectations of asexuality or hypersexuality. These expectations, therefore, lead to restrictions on sexual expression that mandate that individuals with intellectual disability model behaviors that would not be expected by those without disabilities—such as no engagement in sexual activity (de Wit et al., 2022). Similarly, individuals with intellectual disability have historically been left out of conversations about the type of sex education that should be provided to people with disabilities, although there is a recent and growing movement of self-advocates who are contributing their perspectives to this conversation (Friedman et al., 2014).

Media representation of the sexuality of individuals with intellectual and developmental disability shows the integration of both dimensions of epistemic injustice through the reinforcement of persistent stereotypes and myths regarding disability and sexuality. For example, a textual analysis of major newspapers in the USA found that individuals with autism are frequently infantilized, presumed to be unfeeling and disinterested in intimacy, and that autism and romance are oppositional to one another (Brooks, 2018). This type of media representation depicts the limited understanding of people *without* disabilities in regard to the sexuality and sexual experiences of people *with* disabilities. It also prevents those with disabilities from seeing accurate depictions of their experiences in the media, thereby hindering the ability to make sense of their personal experiences.

### Current Study

Those who teach sex education to individuals with intellectual disability are in a unique position to resist or reify epistemic injustices related to disability and sexuality. In the process of educating, they are utilizing hermeneutical rules of how credibility is established that can lend credibility to the learner. Sex educators can also provide interpretative resources so that individuals with intellectual disability can make sense of and communicate their sexual wants and identity. However, sex educators often come from a place of identity-privileged credibility and may not be aware or self-reflexive of their own epistemic ethics.

Given this complex landscape of sexuality and disability, it is imperative to understand how sex educators approach provision of sex education when supporting individuals with intellectual disability. A small body of literature has begun to investigate who is offering sex education to individuals with intellectual disability and what this education looks like in practice (Azzopardi-Lane, 2021; Strnadová et al., 2022). Although researchers acknowledge that people with disabilities face unique challenges to accessing sex education (e.g., Campbell et al., 2020), we still have a limited theoretical understanding of how sex educators who provide instruction to individuals with intellectual disability operate within an environment that exhibits hostility toward the sexual expression of those with intellectual disability. Thus, the current study seeks to examine the experiences of sex educators of individuals with intellectual disability to understand how educators may perpetuate or resist epistemic injustices that individuals with such disabilities face in the context of sexuality and sex education.

This study is part of a broader project examining sex educators’ instructional practices that was designed in partnership with an advisory board of autistic adults (without intellectual disability). It is a standing board that advises on all aspects of the first author’s research program. The advisory board helped us (1) design the scope of the study, (2) develop the interview protocol, and (3) raise awareness of implicit and explicit ableism in our analysis including avoiding ableist language (see also Bottema-Beutel et al., 2021). From this project, we have published composite narratives of service delivery models (Curtiss & Stoffers, 2024) and a thematic analysis of approaches to instruction and assessment (Stoffers & Curtiss, 2024). For this study, we conducted a grounded theory analysis (Corbin & Strauss, 2014) to answer the question, “How do sex educators who teach those with intellectual disability utilize sex education to enable prosocial sexual expression or facilitate the restriction of it?” Grounded theory is a method for constructing theories of social processes inductively from lived experiences thus our answer to this question is in the form of a theoretical model that explains causal and intervening conditions and connections to these conditions to micro and macro contexts (Creswell & Poth, 2018). Although connected to a broader project, this study is a unique analysis.

## Method

### Participants

We used the concept of theoretical sampling to guide participant recruitment. Theoretical sampling is not used to confirm extent theory or constrained by a priori guidelines but rather to a strategy based on concurrent data analysis and collection (Conlon et al., 2020). Thus, we theoretically sampled to maximize opportunities to develop concepts, understand variations within each concept, and hypothesize relationships between concepts (Corbin & Strauss, 2014). To that end, we purposefully recruited adults (over the age of 18) who professionally teach sex education to individuals with intellectual disability in the USA through internet searches, professional networks, and snowball sampling. This yielded 58 sex educators.

We theoretically sampled based on (1) setting of instruction/age served (school, community, clinical, or adult service based), (2) geographic region (states restrictive sex education legislation, states specific mandates to provide sex education individuals with intellectual disability), (3) educational background (special education, public health, applied behavior analysis, mental health), and (4) years of experience/expertise (sex education certification, renownedness). Thus, the participants were able to provide a depth of variation in their experiences with the role of sex education in prosocial sexual expression. We did not theoretically sample based on demographic features and thus there was limited variation on these characteristics. Participants were an average of 40 years old with 9.5 years of experience as a sex educator. Nearly 90% of participants identified as White and 81% of participants identified as female. All but two participants held a college degree and nearly 75% of the sample held a graduate degree. Thirty eight percent of participants exclusively taught adults, 14% exclusively youth, and 48% taught both adults and youth. The majority who taught youth did so starting in adolescence or pre-adolescence, but five educators taught younger children. Education occurred in a wide variety of contexts including K-12 schools, universities, clinics, adult service centers, group homes, and community settings.

### Procedure

We emailed potential participants to let them know of our study, and if they were interested and eligible, they scheduled a time to be interviewed and received an electronic copy of the consent form. All interviews were conducted and recorded via Zoom between June 2020 and October 2020. Once recorded, the interviews, which lasted approximately one hour, were professionally transcribed. Analysis began with the completion of the first interview and lasted until the completion of this manuscript. Analysis was primarily conducted by the authors with some assistance from undergraduate research assistants with whom we met weekly. As we articulated the thematic elements of the analysis, we conducted peer debriefing (Spall, 1998) with other qualitative disability scholars and member checks with members of our original sample who were intentionally selected based on the depth of their original interviews and background variation.

### Interviews

As analysis occurred concurrently with data collection, the semi-structured interview evolved throughout data collection as is consistent with a grounded theory approach (Lassig, 2022). The interview covered topics such as the educator’s background, the resources they utilized, their instructional methods, content-specific instruction (e.g., consent), challenges and facilitators of providing sex education, and educators’ personal values. Some of the questions added in response to the initial analysis included, “Is there anything you think we should ask future participants that we have not asked today?” “How would you teach gender expression?” “How do you incorporate the concepts of consent and vulnerability into your educational practice?” As is consistent with grounded theory interviews, the interview guide served as a starting point but the interviewer modified it to problem-specific areas with follow-up questions, changed the order of the questions, or omitted questions that seemed irrelevant to a particular participant to maximize their unique contribution. The purpose of the interview was to elicit their stories, thoughts, opinions, and conceptualizations (Charmaz & Thornberg, 2012). The interview is a shared space of meaning making in which the participant must interpret their own ideas that we, in turn, interpret again.

### Analysis

We followed Strauss and Corbin and Strauss (2014) guide to grounded theory. Strauss and Corbin do not conceptualize grounded theory as a set of steps or stages but rather provide guidance on how to use analytical tools that allow the researcher to construct a conceptualization of critical context and processes central to the researcher’s question. Through the use of analytical tools, we were able to move from raw data through progressively more abstract concepts to theory. The analytical tools we utilized were asking questions (brainstorming, picking apart, challenging, and extending initial thoughts and ideas when conducting ideas and reading the data), making consent comparisons (comparing data for similarities and differences for the purposes of moving beyond one story to a more abstract level), coding (developing concepts from data and articulating their properties/characteristics and dimensions/variations), exploring concepts through a conditional/consequential matrix (identifying the range of relationships, action, inaction, and emotional responses), memoing (growing in understanding through writing), and diagramming (using visual representations that show relationships between concepts).

Specifically, after we conducted an interview, we would begin analyzing the interview with a process called episode profile analysis—developed by Research Talk and described in Saldaña (2021). First, we read the interview transcript, then we selected approximately ten passages from the transcript that seemed particularly meaningful, then we memoed about these passages, then we memoed about the transcript holistically, then we selected key lines from each passage to create a quote inventory. We then concept coded the key lines which is a method for parsimoniously capturing the essence of line (Saldaña, 2021). We began grouping the concept codes into categories and gave these categories names, definitions, properties and dimensions to create a coding guide. We also sought input on the guide from other experts in disability and qualitative methods (peer debriefing) and a small sub-group of our participants (member checking). The coding guide was used to code the corpus of the data and continued to evolve as the coding took place. Unlike a codebook for which the goal is accuracy and reliability (Braun & Clarke, 2022), we welcomed differential interpretations and confusions about codes, which were how we came to refine and fully define the categories. Throughout the coding process we wrote about interesting features of the data, the codes themselves, and questions we had (memoing). As the coding progressed, we began to identify themes and also diagramed to understand how categories fit together until we discerned we had reached saturation—that we had both incorporated all of our data (coding saturation) and had a robust understanding of the phenomenon (meaning saturation; Aldiabat & Le Navenec, 2018). Thus our analytic process was simultaneously interpretive and systematic.

#### Quality and Rigor Considerations

Considerations of quality and rigor have evolved over time and have taken on a multidimensional conceptualization (Charmaz & Thornberg, 2021). From these conceptualizations (with an emphasis on the strategies that Corbin and Strauss developed over time), we utilized (1) presenting data with analysis so that the reader may assess our conclusions, (2) detailed descriptions, (3) fitness between the theory and the research topic, (4) fitness between the theory and the data, (5) understandability of the theory—even to lay audiences, (6) utility of the theory to bring about change, (7) relevance to the people for whom the theory is about, (8) plausibility of the theory, (9) having key theoretical concepts generated from data, and (10) having variations adequately explained.

We have shown evidence of these elements through the reporting of our methods and findings. Specifically, we used Morse’s (2015) analysis for determining rigor in qualitative research as a guide: rich description, peer debriefing, member checking, audit, triangulation, negative case analysis, clarifying bias, inter-rater reliability, prolonged engagement, and persistent observation. As evidenced by the reporting of our findings, we utilized rich descriptions to justify our analysis. We utilized peer debriefing and member checking (as previously described). We kept an audit trail of all of our coding and decision-making. We utilized two types of triangulation: investigator (multiple investigators examining the data) and data (multiple sources of data for each code). Related to investigator triangulation, any disagreements were discussed until consensus could be reached. Related to data triangulation, through negative case analysis, any instances where data did not fit with our established coding guide were closely examined and caused changes to our analysis. We clarified and challenged our biases throughout the research process and researcher positionality can be found in supplemental materials. We had two slight departures from Morse’s (2015) recommendations. First, Morse discusses the inappropriateness of using inter-rater reliability with unstructured interviews as the interpretive coding process is not designed to be objective. Although we used semi-structured interviews we used this guideline because our coding process was interpretive and there was a great deal of variation in our interviews as our understanding developed—these elements are core features of grounded theory. Second, we interpret prolonged engagement and persistent observation to be applicable to engaging with interview data (as opposed to Morse who indicated that they only apply to observational data) which we achieved as evidenced by our analytic process.

## Results

**Fig. 1:**
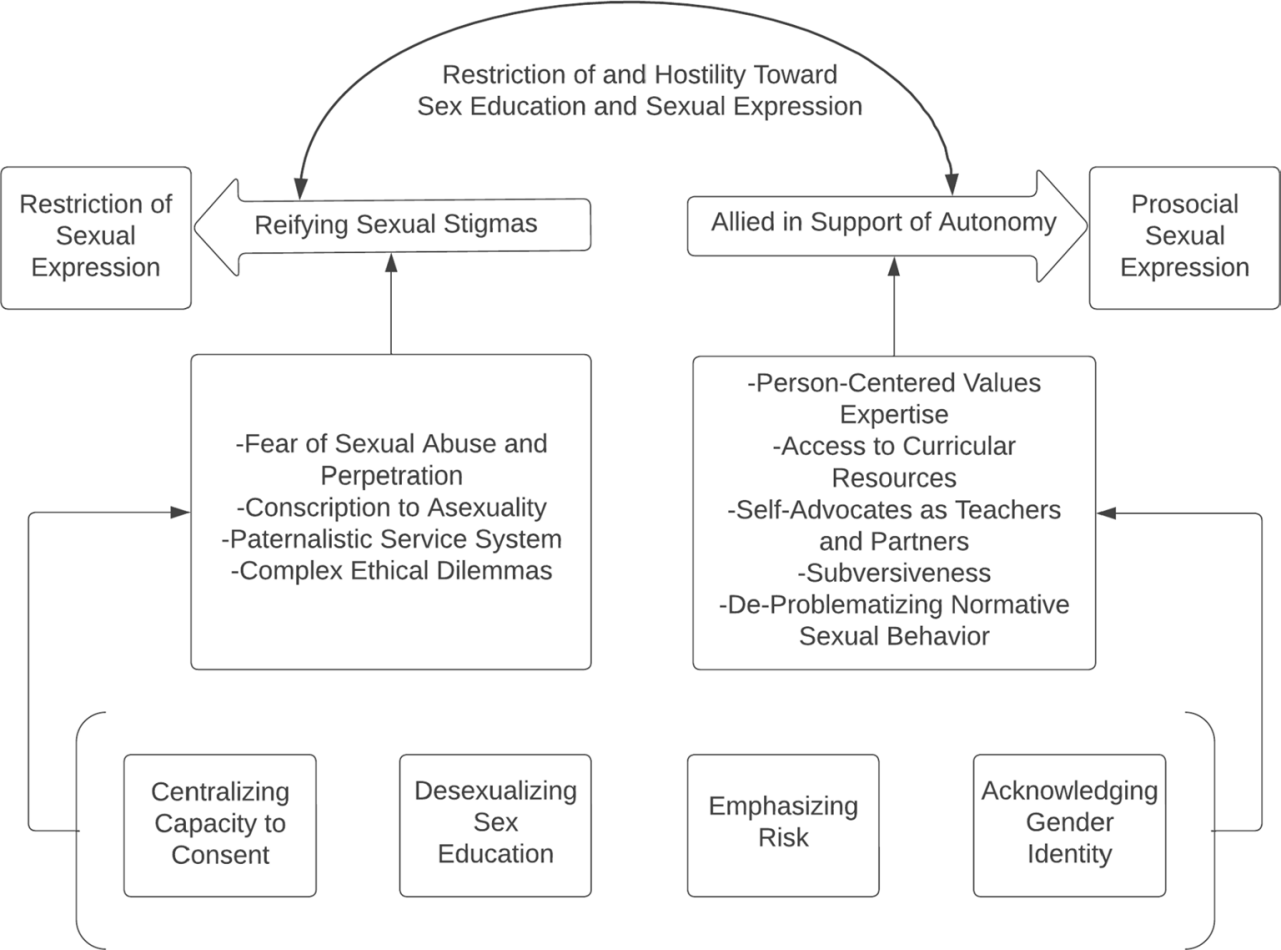
Grounded theory of how sex educators who teach those with intellectual and developmental disabilities utilize sex education to enable prosocial sexual expression or facilitate the restriction of it

The findings of our grounded theory analysis explicate how those to teach sex education to people with intellectual disability both support prosocial sexual expression and perpetuate the restriction of sexual expression. Thus in our theoretical model, these are the outcomes. The process by which educators supported sexual expression was by being allied in support of autonomy—working against hostility in order to advocate for avenues toward sexual expression. Mechanisms that supported educators to be allied in support of autonomy included having person-centered values, expertise, access to curricular resources, self-advocates as teachers and partners, subversiveness, and de-problematizing normative sexual behavior. The process by which educators perpetuated the restriction of sexual expression was reifying sexual stigmas—when their educational efforts contributed to the hostility toward sexual expression instead of dismantling it. Mechanisms that induced educators to reify sexual stigmas included fear of sexual abuse and sexual perpetration, conscription to asexuality, the paternalistic service system, and complex ethical dilemmas. The context in which both of these processes were occurring was the hostile climate towards the sexual expression of people with intellectual disability. First, we will describe the hostility toward sexual expression that educators witnessed. Then, we illustrate the dual and conflicting processes of being allied in support of autonomy and reifying sexual stigmas in four micro-contexts identified by sex educators: centralizing capacity to consent, emphasizing risk, desexualizing (through) sex education, and acknowledging gender identity. Each micro-context provides specific examples of how the mechanisms operate to either promote or restrict sexual expression. See Fig. 1 for an overview of the grounded theory.

### Restriction of and Hostility Toward Sexual Expression

Hostility toward the sexual expression of people with intellectual disability ranged from creating policies that preclude sexual expression (e.g., centralizing capacity to consent to the degree that consent standards are unattainable) to being fearful of sexual contact (e.g., emphasizing risk) to ignoring feelings of intimacy or attraction (e.g., desexualizing) to being unsure that people with intellectual disability were capable of aspects of sexual expression (e.g., acknowledging gender identity). We will discuss each of these ideas in more detail, but we would like to set the stage for the hostility toward sexual expression with a story that an educator shared with us. The story is about love that could have been but was never allowed to grow either because its possibility was never imagined or it was weeded out. Barb, a small business-based educator with 30 years of experience, reflected on a couple’s interaction:

A woman got there and just sat alone by a table, and this guy walked in. It was like there's a million light bulbs on her. She just glowed from ear to ear. The whole rest of the evening, it was like a two-hour thing; the two of them sat at a table like they did not know anybody else was in the room. So, it was just fun to watch them. They weren't inappropriate, they weren't doing anything other than just being very pleased that each other were there. My co-facilitator took her home, and she said, “Listen, I’ve been in your life for 12 years, and I don’t know that I’ve ever heard about this guy. How do you know him?’ She’s like, ‘Well, he’s my friend. He’s my really good friend.’ She said, ‘When was the last time you saw him?’ She said, ‘Two years ago.’ She’s like, ‘Two years ago? He’s your really good friend? When was that?’ Well, he was in the hospital having a procedure, and she could take the bus to the hospital and see him every day…Then when he left the hospital, she didn’t have access to him anymore because she lived with one agency, and he lived with another agency.”

In this story, Barb shares an account of the lack of recognition and respect for emotional intimacy in the lives of people with disabilities. Even as Barb tells the story of heartbreak, she emphasizes the “appropriateness” of the couple’s interactions, implying that “appropriate” is non-sexualized. Elements of restriction were ubiquitous in the educators’ interviews and it was clear that they saw sex education as playing an emancipatory role. They acknowledged that sex education was not a panacea, but our critical exploration will show, like this example, that sex educators played a role in both reifying sexual stigmas and being allied in support of autonomy.

### Centralizing Capacity to Consent

For people without intellectual disability, there are very limited instances when the capacity to consent was called into question (e.g., intoxication), but for people with an intellectual disability, it can be applied much more generally. In each micro-context we explicate, there were instances of being allied in support of autonomy and reifying sexual stigmas. For example, during interviews educators spoke of centralizing the capacity to consent within their instruction, by weaving consent concepts throughout lessons. Some educators, who valued person-centered education, viewed consent as foundational to the autonomy of their students and an opportunity to break down hostilities toward sexuality and disability by providing students tools to engage in prosocial sexual expression. Seth, a school-based educator with three years of experience, expressed the importance of individualization of instruction and supporting sexual expression:

With some creativity there are a lot of different ways that people can communicate consent and be sexual with each other. We support people with disabilities in many ways, like eating and going to the bathroom and going to work and living independently and all these different things. And then when it’s time for sex, it’s like they either have to do it all by themselves, conceptually and perfectly, even though non-disabled people are not like that, or it just can’t happen.

Seth acknowledged the need to defy current norms related to the care of individuals with disabilities, as well as be thoughtful and student-focused in his instruction. He advocated for instruction that would allow individuals the ability and openness to consent to and engage in desired sexual intimacy. Seth pointed out that assistance toward prosocial sexual expression should be as accessible and no different than support provided for other forms of independence. Thus, the mechanism of valuing person-centered education allowed educators to centralize the capacity to consent in such a way that they were allied in support of autonomy and supported prosocial sexual expression.

Sometimes, the topic of consent arose naturally through student conversation, leading educators to manage complex ethical dilemmas and the tension between reifying and challenging sexual stigma. Sierra, a university-based educator with 21 years of experience, described an ethical dilemma in a class where a student reflected on a sexual encounter in high school:

Some girl pushed him in the bathroom, female bathroom, and had oral sex with him. All she said was, “Do you mind if I do this?”... Of course, he didn’t really know what was happening, he was just thrilled that a girl paid attention to him… She did that one or two more times, and then that was it. But he thought they had developed a friendship, that she liked him, and so when he would try to talk to her after these, she would ignore him…At least anecdotally, we know that there are girls who want to be able to perform really good oral sex on their boyfriends and they need to practice, so they practice on somebody with a disability… As much as he still wanted to have the oral sex, he wanted the friendship more. He couldn’t understand…When he was relating this, somebody else in the class… related a similar incident. The two of them, with the support of the rest of the class, got to the conclusion on their own, without me having to say anything, that I think you got used.

This scenario highlighted a complex ethical dilemma in which a student described a time where he was unknowingly exploited and his female peer gained his consent through manipulation. Although Sierra needed to help the students to understand that this situation was manipulative and exploitative, it was also important not to shame the student for engaging in and enjoying sexual activity. However, in Sierra’s retelling, there are moments in which she reifies sexual stigmas, such as when she explains, “Of course, he didn’t really know what was happening.” This phrasing indicates that the educator denied the student’s sexuality and assumed the student was sexually naive. Additionally, while the student certainly has the right to know and understand how this situation was manipulative and exploitative, Sierra did not extend the discussion to helping students to begin articulating what they do want in a relationship, thereby restricting the students’ access to prosocial sexual expression.

While educators sometimes reified sexual stigma during teachable moments, this also extended to planned instruction. In some cases, a paternalistic service system led to restriction of sexual expression. For example, Ashliegh, a BCBA with 11 years of experience, described a new system her agency was preparing to implement to decide an individual’s sex education program:

Each client will have to be assessed for the ability to give consent, and then from there… we have three different curriculums that we’re going to be using across the agency… There will be a curriculum for each group that would be more appropriate to maybe some of the guys. It’s pretty sophisticated stuff that we need to deal with, but maybe some of the other people are like, they just need to know body parts.

In this proposed system, an individual would be assessed on their capacity to consent and tracked into a sex education curriculum believed to align with their ability. This assumes that the student’s current knowledge, as measured on the consent assessment, is stagnant. Therefore, if a student is deemed unable to consent, they cannot receive instruction that might give them the tools to later be deemed able to consent. Additionally, access to curricular topics is dependent on perceived ability level whereby students considered to have less understanding experience greater restriction in accessing sex education instruction and prosocial sexual expression—a level of barrier only experienced by people with intellectual disability.

In many cases, educators discussed the importance of teaching consent due to fear of sexual abuse and sexual perpetration—in fact, Mable, a university-based educator with a year-and-a-half of experience, connected the need to teach consent to a lack of ability to advocate for sexual needs. She said, “I was seeing that a lot of my clients who could not advocate for themselves were getting abused.” Educators felt that students needed to understand expressing and accepting consent to prevent their students from becoming victims or perpetrators. As Reed, a clinical BCBA with one year of experience, described:

It’s highly likely that they’ve [the students] encountered some sort of sexual assaults or inappropriate sexual behavior in the past. And also we know that at a pretty high frequency, the perpetrators of those behaviors are folks in their circle and often peers who also maybe don’t have this robust education. So that consent piece is the biggest one for me, for everybody. But especially their ability to say no and have that honored, and across a wide variety of situations too.

This emphasis on consent as a form of minimizing risk can reinforce common sexual stigmas associated with individuals with intellectual disability. Either the educator is regarding the student as a potential victim and possibly ignoring the students’ sexual desires and need to understand consent as a mechanism for engaging in sexual activity or the educator is positioning the student as a potential perpetrator, drawing upon stereotypes of people with disabilities as hypersexualized individuals whose sexual expression must be restricted.

### Emphasizing Risk

Similar to consent instruction, the fear of sexual abuse and sexual perpetration sometimes led to educational content that emphasized risk and did not fully recognize individuals with intellectual disability as sexual beings. These fears could arise from the educator, the family, or service delivery system, and often led to people with intellectual disability being conscripted to asexuality. Notably, some individuals with disabilities are asexual, but conscription to asexuality refers to unsexing. For example, some educators believed that safety instruction was a top priority and sex education access could be discretionary. Betty, a small business-based educator with 18 years of experience said, “I mean, safety is a core of what I do. I do not push sex education, unless it's something that person really needs. A lot of people don't need it yet, or don't need it right now.” In this quote, because Betty has constricted people with disabilities to asexuality, the only purpose of sex education is as a protective factor. Additionally, the level of access that individuals with intellectual disability had to sex education content could be impacted by caregiver fears, as described by Barb (mentioned above), “We have a lot of aging guardians who strongly believe that they [adults with disabilities] shouldn’t have relationships. So that’s a challenge. Or they think that by putting them in an all male setting or an all female setting, that somehow they’re safer.” This quote also indicates a conscription to asexuality based on caregiver beliefs. Finally conscripting people with disabilities to asexuality can be perpetuated by a paternalistic service system, as Taylor, a public health-based educator with two years of experience, explained:

Our state coalition against sexual assault developed a curriculum that’s supposed to be for folks with [intellectual disability]. I had particular issues with it…It definitely wasn’t coming from a person-centered space of recognizing that all folks have wants and needs and desires and we should be respecting those. It very much came from a little patronizing space. And also it felt very limiting in recognizing the full sexual nature of people. I think it was so focused on, like, if a caregiver is abusing you, here are things that you can do instead of also focusing on like, here is how you practice healthy relationships in your life and assert your boundaries.

All three of the above quotes demonstrate how decisions surrounding sex education content are often rooted in the fear of sexual abuse and sexual perpetration and based on people with disabilities being conscripted to asexuality. In each of these quotes, the educator, caregiver, or service system prioritized safety over sex education, which emphasizes risk to the student and could inhibit healthy sexual expression, as well as reify stereotypes that all individuals with disabilities are disinterested in sexual experiences and therefore can only be victims of sexual abuse. As was highlighted by Taylor, safety instruction in isolation does not offer individuals the opportunity to learn strategies for prosocial sexual expression. Thus, an emphasis on risk can narrow the lens through which sex education instruction is offered.

The extreme form of narrowing the lens through which sex education is offered was exemplified by educators who worked with perpetrators of sexual violence. This presented a complex ethical dilemma in which the emphasis on eliminating the problematic behavior outweighed support with prosocial sexual behavior, as indicated by Reed (mentioned above),

We definitely have folks seeking out support around sexual behavior that’s going to be harmful to others, or potentially incur legal harm for themselves. So, anything that involves not having clear discrimination between what’s going to be acceptable in public versus private. A lot of folks that have trouble with age discrimination. So, maybe some approaching behaviors that aren’t even necessarily super sexualized, or maybe sometimes they are, but are being directed at folks where it has the appearance of being problematic.

Reed clearly explains the problematic behavior (“approaching behavior directed at folks where it has the appearance of being problematic”) and the educational solution (teaching age and/or privacy discrimination). However, given the ethical complexity, support for prosocial sexual behavior is minimized. On the other end of this complex ethical dilemma, educators had to teach in the context of trauma as many of their students had experienced sexual abuse. Matt, a disability program-based educator with one year of experience, discussed sexual trauma and the emphasis on risk in his instruction, “We’re pretty adept at talking about trauma, and abuse, and things like that, because that tends to come up—especially with this population. So we’re not as apprehensive toward it. The topic is there, and it’s important, because that goes with consent and advocacy, and things like that.” Although we did have educators that emphasized the risk of sexual abuse inherent in a paternalistic service system, the emphasis of instruction was almost always on the individual, not on addressing the service system.

Unfortunately, given the myths surrounding the sexuality of individuals with disabilities, educators often reported that access to funding mechanisms in a paternalistic service system required an emphasis on risk. Funding agencies might not view prosocial sexual expression as an important goal in the lives of individuals with disabilities. This was explained by Abi, a disability program-based educator with three years of experience:

We did not receive the grant, and when I called just to get a little bit more information about why, because it’s a yearly grant, they told us this wasn’t a priority when it came to funding things for folks with developmental disabilities. To which I said, “Okay. Thank you.” And hung up the phone. I think there is definitely the public perspective of like, folks with [developmental disabilities], they don’t care about sex.

For Abi, funding was denied for comprehensive sex education services because this was not deemed important to the lives of individuals with intellectual disability. Other educators reported that taking a risk prevention perspective led to funding opportunities via organizations that valued sexual abuse prevention such as Taylor (mentioned above):

So we get money from the CDC. It is a subset of VAWA—the Violence Against Women Act. It is particularly RPE money, which is Rape Prevention Education money. And that gives us a pretty broad spectrum [to] provide services to our service area. It isn’t restrictive in terms of age or particularly what your content covers. As long as we can justify [that] the things we talk about will lead to a—hopefully—prevention or reduction of sexual violence, we can talk about it.

With these funding streams, educators can attempt to tie in aspects of prosocial sexual expression and comprehensive sex education, but they also must connect back to abuse prevention, which could unnecessarily emphasize risk within instruction, restrict access to sexual expression, and reify sexual stigmas.

Given that community stakeholders and funders were more comfortable granting individuals with disabilities access to instruction emphasizing risk, educators sometimes reported the need to demonstrate subversiveness. Some educators used safety instruction as a way to get their foot in the door, but then offered more comprehensive sex education services once they gained access to the students and/or the necessary resources.

Morgan, a disability program-based educator with six years of experience described how this type of subversiveness could get caregivers and service providers to buy-in to instruction:

So, I was able to go in and say, like, “Now, I have this new sex ed program.” That was a little bit of a more difficult sell for some teachers—certainly in some schools. But again, we’ve always framed it around safety and abuse prevention, and that’s been a good way for us to get into schools, and also to talk to parents.

Morgan suggests that by addressing safety needs and emphasizing the risks students face, schools and caregivers were more likely to consider and accept the importance of sex education instruction—rather than if the educator emphasized prosocial sexual expression. Paradoxically, this approach can reinforce myths related to the sexuality of individuals with disabilities; however, it also allows the educator to gain access to students and resources and utilize this access to provide comprehensive sex education instruction.

Importantly, self-advocates as teachers and partners recognized instruction emphasizing risk and vulnerability could lead to limitations on information that reduces risk. Brittany, a university-based educator with 12 years of experience who also identified as a self-advocate, suggested that intentions to protect individuals with disabilities often created greater risk:

Basically, trying to protect people does not keep them safe. It makes them at a higher risk. Some people like to use the V word. Do you know what the V word is? Vulnerable. They like saying people with disabilities are vulnerable. I don’t like that word. The only reason we’re more vulnerable is because people don’t give us the information. In my work, I don’t use the word vulnerable.

Brittany highlights the idea that positioning people with disabilities as vulnerable can insinuate they are easy victims and they should be fearful of society and sexuality which echoes the work of scholars who have emphasized the structural aspects of sexual violence (e.g., Curtiss & Kammes, 2020; Hollmotz, 2009). Brittany asserts that when given the appropriate information to increase safety, individuals with disabilities can engage in safe practices that also allow space for prosocial sexual expression.

Many educators recognized that risk is an inherent part of life for all individuals and people have the right to make decisions that carry some risk. As Jasmine, a disability program-based educator with two years of experience explained:

I’m a believer in the whole idea of dignity of risk… If you want to be promiscuous that’s fine. But having some balance of you having the right to do this, this is enjoyable, you want to do this, then also weighing the potential risk of that. And teaching both of those things. I think that’s our responsibility—saying, “No, sex does not mean love. It can. But it does not inherently mean that.” And it might be risky to have sex with someone that you don’t want to marry or whatever the situation.

Jasmine expressed her belief that it is the right of the individual to accept risk when they have the information and knowledge to understand the level of risk they are accepting by making a choice. It is, therefore, the sex educator’s job to help individuals understand the risks and navigate balancing risk with desire.

Finally, educators allied for the autonomy of their students recognized that, sometimes, a double standard for people with disabilities existed related to risk, as Meradith, a public health-based educator with five years of experience explained:

And like, God forbid, someone makes a mistake in a relationship, right? Like we all have made mistakes in a relationship. I think that so often folks with disabilities are protected from making any mistake. And if you don’t have a chance to make mistakes, you don’t get to learn from your mistakes. So there’s this concept of dignity in risk, right? And being allowed to take risks… And yes, you have the right to make decisions for yourself.

Like Jasmine, Meradith is emphasizing the importance of dignity in risk: we all have the right to make mistakes. Meradith explains that, while individuals without disabilities are frequently allowed to make mistakes in relationships with minimal supervision or judgment, individuals with disabilities are often protected or restricted from making mistakes. Yet, mistakes offer learning opportunities and lessons. Allowing individuals to make their own choices, whether these choices include risk-taking behaviors or not, is a human right.

### Desexualizing (Through) Sex Education

Like emphasizing risk, desexualizing sex education was another strategy that educators used to provide sex education, given the hostile environment toward sex education and the paternalistic service system. For example, Natalie, a small business-based educator with twenty years of experience said:

And so here’s a group of people that didn’t have any kind of sexuality education. They’re now working in a work site and never had any education around what’s okay to do in the work site around relationships and sexuality. So we took the sexuality education curriculum and cut it down to really focusing on healthy work relationships.

In this excerpt, Natalie explains how she had to edit her curriculum to exclude sex and focus on healthy relationships to be able to teach “sex” education. Natalie links desexualizing sex education to desexualizing through sex education as she was brought in to provide instruction only when a person had inappropriate sexual behavior, as shown in this statement:

And the reason [the employment program] reached out to me is that many people were losing their jobs. Not because they couldn’t do the actual job, but because they were asking coworkers out or hugging someone they weren’t supposed [to], those sorts of healthy work boundaries.

Natalie was asked to teach “sex” education for the specific purpose of using education to teach “healthy” sexual boundaries, such that there were no realistic avenues for sexual expression—conscripting the person to asexuality. She indicated this when she said, “How do you move from a friend to a partner? And who is it okay to be in a relationship with? It’s not your coworker, it’s not your boss.” This statement does not recognize how many people without disabilities form romantic and/or sexual relationships in the workplace. Additionally, families and service providers are rarely providing alternative avenues for building those relationships—work may be the only place where they meet other people.

Natalie illustrates the two sides of Desexualizing (Through) Sex Education: (1) when sex education lacks instruction on physical intimacy and (2) when sex education is used to address problematic sexual behavior. Regarding the lack of instruction on physical intimacy, some educators intentionally desexualized their instruction as an act of subversion in order to improve access to some level of sex education. For Morgan (mentioned above):

Usually, the way that we try to work it is, we say, “Well, can they stay for these topics?” They’re like, “Yeah, healthy communication is fine. Healthy decision-making is fine, but I don’t want them to do types of sex.” So, those different negotiations, so that we can at least have the students in some of the topics.

In this example, Morgan has a preset curriculum, and she negotiates with parents to allow students to attend at least some sessions. Even when attempting to be subversive, this may inadvertently reify sexual stigma as it may be interpreted as a validation that sexual information is unnecessary. Another way sex education lacked instruction on physical intimacy was not due to the hostility toward sex education but because of the educator’s own lack of expertise or access to curricular resources. For example, Amber a disability program-based educator with one-and-a-half years of experience said:

Obviously, one of the things that I teach people is different forms of sex, so literally, vaginal, oral, anal, things like that. And I think I would like to know how to provide more resources or education to people around how—when I teach that, it’s very surface level…I think I would like to access or find more resources on providing different things about pleasure, sexual pleasure, for people with intellectual, developmental, and also physical disabilities and how that may look different for people depending on their disabilities.

The educator had a willingness to teach about physical intimacy but was unsure how to ensure it is inclusive of people with disabilities. Similarly, Stephanie, a disability program-based educator with six years of experience, commented on the lack of curricular resources,

I think all of the stuff I mentioned before about pleasure and about normalizing difference and normalizing kink and fetish and making it adult. So many of the things I’ve seen are not adult in nature. It’s sort of like, so we’re teaching puberty, but this person’s 45. And I get that that’s the only sex ed curriculum you had—to get to puberty was like lesson number three. But really, we’re teaching this 45-year-old man about getting menses.

Stephanie also specifically connected how the lack of sex education with detailed instruction on physical intimacy interacted with the paternalistic service system:

I think we miss out on pleasure. I think we miss out on BDSM, fetishes, kink. There is some research out there that shows that people who don’t have access to a “normalized sexual experience,” will get super creative with how to have that. So if you live in an environment where you are watched 24 hours a day and supervised in all these different things, and the only private time you have [is] in a bathroom - the way you masturbate might get funky… And I don’t think we normalize that enough to anybody, somebody with a disability or not.

Stephanie’s quote highlights how, in a heteronormative society, some forms of sex are seen as more acceptable than other forms of sex and suggests that educators need to de-problematize normative sexual behavior. Similarly, Brook, a small business-based educator with 37 years of experience discussed a situation in which she was asked to consult with a young woman who was found in the park not wearing a shirt. The young woman’s direct support staff thought she needed help understanding privacy and boundaries; however, in talking to the individual, the educator found that the young woman was with her boyfriend in the park because they had no other place to be intimate. Brook commented that in this case, it was not a misunderstanding about privacy, but rather, “This issue then became, how are you supporting people in normal, healthy dating relationships here?” In this situation, Brook was able to challenge the providers’ policies regarding sexual expression by valuing person-centered education. Brook was able to show the provider how “the lack of a policy or a support menu or map was what actually created the inappropriate social behavior.” In this case, the lack of a policy or support menu were concrete examples of the paternalistic service system.

Brook (and to some extent Stephanie) allude to the idea that restricting prosocial sexual expression and sex education can cause problematic sexual behavior. Educators also discussed how sex education was used to address problematic sexual behavior, often in a way that was also restrictive of prosocial sexual expression. Vanessa, a public health-based provider with three years of experience, discussed the reactive way she is asked to provide sex education:

Nine times out of 10, it’s reactive and not proactive. So instead of…whoever is getting to an age where they might start becoming interested in sex and relationships, it’s always, “They did this. I caught them looking at this. They engaged in this behavior. They asked this question.” Then the support person or parent is freaking out.

Vanessa’s passage emphasizes that sex education instruction is being used as a tool to try to eliminate sexual behavior. Similarly, Paul, a clinical BCBA with eight years of experience talked about being asked to provide education specifically to reduce sexual behavior that highlights people with disabilities’ conscription to asexuality,

So online hookups, oh my gosh. That’s one of the biggest areas I get feedback. Like, “Oh, they can’t handle this, or that’s not safe.” And I’m like, “It’s not safe for any of us. Let’s be real. It’s not safe.” The idea that I’m safe if I hook up online and go to a stranger’s house is just as absurd as the idea that my client would be safe going to a stranger’s house. You don’t go to strangers’ houses, but we do. That’s become part of our cultural sexuality. So if other people can have access to this and my client is capable of recognizing and gaining consent and is capable of recognizing and providing assent, why shouldn’t they be able to learn this?

In this example, Paul is subversive and de-problematizes normative sexual behavior despite pressure to provide “sex” education without a realistic pathway toward sexual expression. Similarly, Ashliegh (mentioned above) discussed how she worked with a women who had experienced sexual assault to feel safe in her intimate relationship,

I work with [someone who] has a history—she was sexually assaulted and has always been very afraid of this stuff, but she’s been dating this guy and now she’s engaged and they want to have an intimate relationship. We’re doing a lot of work with not only her, but also her fiance, he’s his own guardian, so he can come in and we’re doing advocacy between the two of them. Helping her out to use, we call it “yes, no, fast, slow.”

Ashleigh provides a counterexample of how when sex education centralizes sexual intimacy educators can be allied in support of autonomy. The final theme, acknowledging gender identity, provides other examples of how educators were allied in support of autonomy.

### Acknowledging Gender Identity

We directly asked educators what areas they saw as being gaps in the education they provided, and gender identity was a common response. We also directly asked some educators how they would teach gender identity or gender expression, and several said that they did not teach gender identity or struggled to, such as Mable (mentioned above):

I’ve only done one lesson, and I only had three participants in it. It wasn’t the most successful course that I ran. I think some of the information, just given the three clients I had in that particular session, seemed to be a really hard topic for them to grasp. And I think it’s because it was the first time they were hearing any of the information. And I mean, even for me, I’ve heard it, a dozen to a 100 times, and there’s still areas where I’m like, “Am I using the right pronoun?”

Mable touched on hesitation—either on the part of participants or their parents—with gender identity, as well as her own struggle with the topic and her ability to teach it. The implication was that after the initial poor response, she chose not to teach gender identity again, thereby restricting students’ access to instruction supporting sexual expression.

Yet, a different educator, Marie, a university-based educator with five years of experience, who had more expertise and self-advocates as teachers and partners, did not struggle to teach gender identity. Marie leveraged her expertise and partners to get a better understanding of what individuals with intellectual disability wanted to learn and the educator developed multiple resources (this passage focuses on videos):

I’ve tried to get more into videos and things like that, that’s something that folks told me they really liked a lot. We actually developed two videos with actors and actresses with disabilities. One was on sexual identity and gender identity… It was a really great dynamic that was going on, and I anecdotally felt like they were going to take something away from that. Then there was another student who was still very confused about what we were talking about, and so we would try to break it down differently. That’s where some of those other ways of teaching things would come into play.

Like Mable, Marie had a situation where she struggled to teach gender identity, but she relied on their own expertise and curricular resources to overcome that challenge. Several educators discussed how discussions of gender identity were embedded within their curricula, for example, Bailey, a public health-based educator with five years of experience said,

So, we actually have a whole presentation on identities. We talk about biological sex or what we call “sometimes body parts,” and then we talk about gender identity, we talk about gender expression, and we talk about sexual orientation…we often ask students how they express themselves and reminding students that it’s really important to be really respectful to the way that other people express themselves. We can assume someone’s gender identity or their sexual orientation by the way that they’re expressing themselves.

This example illustrates how some educators embed gender identity into instruction and also how doing so is a reflection of the value that all gender identities and sexual orientations should be validated and respected. We did have some educators who explained that either their client or their client’s guardian did not share that value, for example, even going so far as to ask for conversion therapy in Reed’s (mentioned above) experience:

It’s also been really idiosyncratic whether [gender identity] is directly planned into their programming or not because that also is a safety concern. And so for me, I find far more frequently that this isn’t something that folks are necessarily approaching when they seek out services with us—unless they’re specifically, we have had many folks seeking conversion therapy for their clients. So assuming that we might engage in conversion therapy and then that might be part of what we do, which clearly it’s very antithetical to our ethos and values and work that we do, but if it’s not that, I’ve found that at least for me and my clients that I’ve seen, it hasn’t come up until later and has been self-disclosure if it’s come out in sessions.

In this example, this educator’s most salient experience with gender identity is with people seeking conversion therapy (which Reed is clear that they do not provide). Reed also indicates gender identity is not something that would be taught unless it “came up” in sessions. The discrepancy between interpreting gender identity as a topic that should universally be taught or taught only if it “came up” seemed to depend on the educator’s expertise, partnership with people with disabilities, access to curricular resources (as already mentioned) as well as the educator’s interpretation of valuing person-centered education. In this example, Reed does not provide education on gender identity without a clear indication the individual wants or needs that education, but that thinking assumes (1) all individuals already have the ability to communicate what information they want and (2) that education in itself does not have a role in helping individuals find information they need. Thus even though Reed was trying to be person-centered, this educator was actually suppressing sex education.

Valuing person-centered education is not limited to only providing education that individuals ask for, but also providing access to information they would be able to obtain on their own if there were not so many barriers to instruction. Amber (mentioned above) explained the necessity of providing a baseline of education:

I really, really try to stress to folks that it’s—a lot of what we’re talking about, and what they’re learning—is 100% normal and okay. Not only is it normal to talk about, but it’s also normal to have variations in body parts, in sexual orientations, and in gender identities. That’s super important to me. I have definitely had negative reactions from some providers or people without disabilities asking why it is that their people with disabilities need to learn things like sexuality and gender because I’ve had people just assume that not only are people with disabilities asexual and that they don’t ever question their gender and sexuality.

In this comment, without introducing gender identity, Amber has found that there is not necessarily an opportunity to discuss normal variations, but that it is a topic that applies to everyone—not just those with non-heteronormative identities.

Furthermore, by setting the stage that gender identity is a normative topic, it opens the door for educators to be allied in autonomy when individuals need gender-affirming care, as evidenced by Jacob, a disability program-based educator with four years of experience:

I think that goes back into the whole if we don’t talk about it, it won’t happen thing. But, the thing is just realizing that it’s a thing for everybody. I mean, just because you have an intellectual disability or something of that effect doesn’t mean that you can’t feel like, “Hey, I really ... I’m a guy, but I feel more comfortable as a woman.” That happens. Actually, one of our members is in the process of transitioning right now. I think there’s that fear of, well, if you teach it to them, they’re not going to understand it fully. They’re going to go off and get involved with it, and it’s not really a thing.

Jacob goes on to discuss the ways in which the organization supported this person throughout her transition. This passage also illustrates the connection between paternalism in sex education and the paternalistic service system more generally—that leads those in power (paid staff who do not have disabilities) to be patronizing of the wants and needs of those with intellectual disability.

## Discussion

Through our grounded theory analysis of interviews with sex educators, we identified the context under which sex education is provided (hostility toward the sexual expression of people with intellectual disability). The hostility toward sexual expression exists within a broader context of lack of standardization of instruction, and often, broad hostility toward sex education that generally exists in our society and especially for people with intellectual disability. We then explored how educators were allied in support of autonomy or reified sexual stigmas through four micro-contexts. Each area presents a unique example of potential epistemic injustice. Within each area, there were multiple mechanisms that influenced how sex educators who teach those with intellectual disability utilized sex education to enable prosocial sexual expression or facilitate the restriction of it.

*Centralizing capacity to consent* presented an example of testimonial injustice to the degree that neither people with intellectual disability assertions of sexual desire nor experiences of abuse were believed. The law in this area is complex as each state sets its own guidance in determining the capacity to consent, and judicial case law in this area may be lacking (Linder, 2018). Furthermore, laws on the capacity to give consent are not always congruent with a state’s guardianship laws which often presume guardians to have the authority for all reproductive health decisions, including consent (Lanier, 2019). As sex educators enter this complex legal landscape, some resisted this injustice by focusing on enhancing decision-making capacity, which is consistent with other studies (e.g., Dukes & McGuire, 2009), thus using their own hermeneutical power to increase the credibility of those with intellectual disability.

Educators also emphasized risk as a hermeneutical strategy to improve access to sex education. In doing so, they may perpetuate hermeneutical injustice such that the lived experience of those with disabilities was constrained by narratives of abuse. Over twenty years ago, researchers were bringing attention to missing discourses of pleasure regarding sexuality and disability (Tepper, 2000) and pleasure continues to be omitted from sex education (Martino, 2024; Turner & Crane, 2016). In their systematic review of sexuality-based intervention programs for those with intellectual disability, Black and Kammes (2021) found that interventions are increasingly focused on relationship development, but the majority have focused on abuse prevention. Desexualizing (through) sex education was a similar strategy that educators utilized to improve access to sex education, which could also constrain the sexual expression of individuals with disabilities. Martino (2024) discusses a form of sexualizing (through) sex education in the form of medicalization. In Löfgren-Mårtenson’s (2013) exploration of intellectual disability and sexual scripts, she theorized the “restrictive script” (p. 417) as how culture and values are influenced by normativity such that the presence of any type of sexual expression by people with disabilities is perceived as deviant.

According to Löfgren-Mårtenson (2013), the restrictive script is based on heteronormative values such that those with disabilities with non-binary conforming identities experience the most stigma. This is similar to how Martino (2024) discusses the contraction of good/healthy sexuality. In our study, acknowledging gender identity was a way that educators resisted epistemic injustice by dismantling restrictive scripts. In their review of social inclusion and gender-diverse adults with intellectual disability, Smith et al. (2022) found a lack of support, the persistent presence of homophobia and transphobia among care providers, and a lack of inclusive sexuality education. Similarly, autistic transgender and non-binary adults have discussed difficulties with healthcare and support professionals (Bruce et al., 2023).

Being allied in support of autonomy allowed educators to resist epistemic injustice. Across the themes, the mechanisms that supported educators to be allied in support of autonomy included having person-centered values, expertise, access to curricular resources, self-advocates as teachers and partners, subversiveness, and de-problematizing normative sexual behavior. Several of the mechanisms we identified for being allied in support of autonomy are consistent with the idea of cripping sex education—which is the application of crip theory to sex education (Martino et al., 2024). Crip theory evolved from queer theory and is a critique of mindbody normativity (McRuer, 2006). When applied to sex education it calls for sex education to broaden understandings of sex and sexuality, center the voice of disabled people, and disrupt ableism and heteronormativity embedded in sex education (see special issue of Sex Education edited by Campbell et al. (2020) for a full review of cripping sex education).

Having person-centered values was expressed when educators highlighted the importance of individualized instruction, challenged providers’ policies, and provided an education that was accessible to those without disabilities. *Expertise* was a salient factor in educators feeling comfortable teaching specific topics and worked in conjunction with access to curricular resources such that those with greater expertise could either find or make the curricular resources they needed. This is unsurprising, given that many educators report a lack of training related to sex education and struggle with feelings of competence in providing sex education (Goli et al., 2022). However, when provided with training and support, educators experience enhanced teaching readiness and knowledge-seeking (Curtiss & Ebata, 2016).

Educators who had self-advocates as teachers and partners or were self-advocates themselves were often pushed to be more allied in support of autonomy by these partners, such as when self-advocates highlighted emphasizing risk could limit access to information or pushed educators to develop more expertise in how to teach specific topics. Self-advocates have expressed these concerns in prior literature: such as reporting a greater need for instruction to address positive sexuality and minimize the focus on risk (Campbell et al., 2020; Hole et al., 2022; Martino, 2024). Educators used subversiveness in order to get caregivers and service providers to buy into sex education instruction and provide more realistic instruction that acknowledged sexual expression as a goal of instruction. De-problematizing normative sexual behavior was often necessary given a heteronormative society that sees certain types of sexual expression as deviant, any sexual expression of people with disabilities as inappropriate, and only acknowledges gender on a binary. Prior literature has reported that working with caregivers as a part of a sex education intervention can help caregivers to normalize sexual behaviors (e.g., masturbation) rather than view such behaviors as problematic (Chou et al., 2019). Consistent with addressing hermeneutical injustice, the normative sexual behavior of people with disabilities should be de-problematized broadly in society as it contributes to the hostility and stigma experienced by people with disabilities.

There were times when educators facilitated the restriction of sexual expression and perpetuated epistemic injustice through reifying sexual stigmas. Across the themes, the mechanisms that induced educators to reify sexual stigmas included fear of sexual abuse and sexual perpetration, conscripting to asexuality, the paternalistic service system, and complex ethical dilemmas.

Sexual abuse is a significant concern for people with intellectual disability as it is estimated that they may experience ups to seven times the rate of sexual assault compared to those without disabilities (Byrne, 2018; Shapiro, 2018). However; educators’ fear of sexual abuse and sexual perpetration could reify sexual stigmas when the focus of instruction was to understand strategies for expressing and accepting consent when consent is synonymous with saying “no” to sexual activity. Although individuals with disabilities are at greater risk of sexual abuse, and should receive instruction on the legal aspects of sexual expression in private life and the workplace, an individual’s vulnerability is often emphasized without the broad contextual factors that contribute to increased susceptibility (Curtiss & Kammes, 2020). The fear of sexual abuse and sexual perpetration worked in tandem with conscripting to asexuality. We use the word conscription to differentiate this mechanism from having an asexual sexual orientation. Conscripting to asexuality occurred when education was used to reduce sexual behavior and was reinforced by the paternalistic service system. The paternalistic service system was indicative of the policies, laws, and funding mechanisms that restrict opportunities for the sexual expression of people with intellectual disability. Foley (2017) has theorized that the service system for those with disabilities is often characterized as a “paternalistic regime” (p. 6) in which people with disabilities are denied control over their own lives. Finally, educators faced complex *ethical dilemmas* that could create ambiguity about how it was possible to avoid reifying sexual stigmas, especially in the context of sexual abuse and perpetration.

Our research has several limitations that are important for us to acknowledge. First, although we have theoretical saturation for many service models of how sex education is provided, we had very few educators who provided sex education embedded in schools (we had many who provided sex education in schools on a contractual basis). These educators are an important service delivery model that is under-explored in our analysis, as are educators from culturally and linguistically minoritized backgrounds. Additionally, our grounded theory only reflects the point of view of sex educators. The views of individuals with intellectual disability, parents, and service providers are absent. Although there is some previous research (e.g., de Wit et al., 2022; Hole et al., 2022) in these areas, it remains an important area for future research.

Our research provides insights into mechanisms that educators have and can use to be allied in support of the autonomy of individuals with disabilities to enable the sexual expression of people with disabilities. Simultaneously, we also unpack how sex education can potentially reify sexual stigmas and cause additional barriers to sexual expression. The mechanisms we identified can be further explored in the literature and utilized in trainings for current and future sex educators. It is not sufficient for individuals with intellectual disability to have access to sex education. They need access to sex education that enables them and that, in and of itself, challenges the hostility to the sexual expression of people with disabilities.

## Supplementary Information

Below is the link to the electronic supplementary material.

Supplementary file1 (DOCX 7 KB)

## Data Availability

Due to the sensitive nature of the data they will not be shared in its raw form.

## Code availability

A coding audit is available upon request.
