# Supplementary material for: Theorizing the Role of Sex Educators in the Resistance and Reification of Epistemic Injustices Related to the Sexual Expression of People with Intellectual Disability
Source: Arch Sex Behav. 2024 Nov 14;54(2):605–21. doi: 10.1007/s10508-024-03039-5 (PMC11835894; doi:10.1007/s10508-024-03039-5)
Supplement: Supplementary file 1 — Supplementary file1 (DOCX 7 KB) [file 10508_2024_3039_MOESM1_ESM.docx]

**Researcher Positionality**

The first author taught sex education to individuals with intellectual and developmental disabilities for six years when she worked in a disability program. During that time, she oversaw the organization's sex education services, trained other direct service providers to be sex educators, and was a liaison with the Office of Inspector General to prevent, identify, and investigate abuse. During her graduate program, she continued to teach sex education to autistic youth and adults and provided state-wide trainings. She has continued conducting research in the area of disability and sex education since that time. The second author served as a public school special educator for eight years, working with elementary and middle school students with disabilities. Although she has worked with students with a diverse range of abilities in school settings, the second author was never responsible for providing sex education instruction to students. During her graduate program, she has collaborated with individuals with and without disabilities to develop accessible and inclusive sex education content, and has conducted research related to disability and sex education.
